# Expanded nursing roles to promote person-centred care for people with cognitive impairment in acute care (ENROLE-acute): study protocol for a controlled clinical trial, process and economic evaluation

**DOI:** 10.1186/s12877-023-04560-3

**Published:** 2023-12-14

**Authors:** Verena von der Lühe, Marcelina Roos, Mareike Löbberding, Nadine Scholten, Wiebke Müller, Martin Hellmich, Dusan Simic, Sascha Köpke, Martin N. Dichter

**Affiliations:** 1 University of Cologne, Faculty of Medicine and University Hospital Cologne, Institute of Nursing Science, Gleueler Straße 176-178, Cologne, Germany; 2 University of Cologne, Faculty of Medicine and University Hospital Cologne, Institute of Medical Sociology, Health Services Research and Rehabilitation Science, Chair of Health Services Research, Eupener Straße 129, Cologne, Germany; 3 University of Cologne, Faculty of Medicine and University Hospital Cologne, Institute of Medical Statistics and Computational Biology, Robert-Koch-Straße 10, Cologne, Germany; 4 University of Cologne, Faculty of Medicine and University Hospital Cologne, Institute of Health Economics and Clinical Epidemiology, Gleueler Straße 176-178, Cologne, Germany

**Keywords:** Person-centred care, People with cognitive impairment, Expanded nursing roles, Advanced Practice Nursing, Hospital, Complex intervention, Study protocol, Controlled clinical trial, Process evaluation, Economic evaluation

## Abstract

**Background:**

For people with cognitive impairment, hospitalisation is challenging and associated with adverse events as well as negative outcomes resulting in a prolonged hospital stay. Person-centred care can improve the quality of care and the experience of people with cognitive impairment during hospitalisation. However, current care processes in German hospitals are rarely person-centred. To enable successful implementation of person-centred care on hospital wards, change agents within the interprofessional team are key. The aim of this study is to test the feasibility and initial effects of a newly developed complex person-centred care intervention for people with cognitive impairment provided by expanded practice nurses in acute care.

**Methods:**

We will conduct an exploratory non-randomised controlled clinical trial with accompanying process and cost evaluation with three intervention and three control wards at one university hospital. The person-centred care intervention consists of 14 components reflecting the activities of expanded practice nurses within the interprofessional team on the intervention wards. The intervention will be implemented over a six-month period and compared with optimised care on the control wards. We will include people aged 65 years and older with existing cognitive impairment and/or at risk of delirium. The estimated sample size is 720 participants. The primary outcome is length of hospital stay. Secondary outcomes include prevalence of delirium, prevalence of agitation, sleep quality, and person-centred care. We will collect patient level data at six time points (t_1_ admission, t_2_ day 3, t_3_ day 7, t_4_ day 14, t_5_ discharge, t_6_ 30 days after discharge). For secondary outcomes at staff level, we will collect data before and after the intervention period. The process evaluation will examine degree and quality of implementation, mechanisms of change, and the context of the complex intervention. The economic evaluation will focus on costs from the hospital’s perspective.

**Discussion:**

The ENROLE-acute study will provide insights into the effectiveness and underlying processes of a person-centred care intervention for people with cognitive impairment provided by expanded practice nurses on acute hospitals wards. Results may contribute to intervention refinement and evidence-based decision making.

**Trial registration:**

Current controlled trials: ISRCTN81391868. Date of registration: 12/06/2023. URL: https://doi.org/10.1186/ISRCTN81391868

**Supplementary Information:**

The online version contains supplementary material available at 10.1186/s12877-023-04560-3.

## Background

In 2021, almost half of patients in German hospitals were 65 years and older [1]. Older age is associated with cognitive impairment, with a prevalence among elderly hospital patients ranging from 40 to 67.5% [2, 3]. This mainly includes people living with dementia, mild cognitive impairment, and with delirium [3].

Hospitalisation of people with cognitive impairment is associated with several challenges. One challenge is a frequently missing formal diagnosis of cognitive impairment [2, 3], resulting in care that is not tailored to the needs of this vulnerable patient group. An integrative review on hospital outcomes [4] found, that people with cognitive impairment encounter a hospital environment focussed on acute illness rather than their individual needs, as shown by avoidable bed moves, inadequate pain management, inappropriate catheterisation, and poor communication and relationship building with healthcare professionals. Negative experiences are common, frequently leading to feelings of fear and an increase in behavioural and psychological symptoms. In addition, people with cognitive impairment are more likely to encounter a range of related adverse events such as the incidence of delirium, urinary tract infections, pneumonia, pressure ulcers, adverse drug reactions, and falls, that may lead to prolonged hospital stays and increased mortality [4]. Healthcare professionals struggle with the discrepancy between desirable hospital care and the current reality of care for people with cognitive impairment, resulting in distress and frustration, uncertainty in dealing with changed behaviour, conflicting priorities, and the inability to meet individual needs [5].

A key to improve hospital care for people with cognitive impairment is the concept of person-centred care (PCC), which has been recommended in guidelines [6, 7] and by representative organisations [8]. Person-centredness has been applied to different target groups such as people living with dementia [9], different healthcare professionals such as nurses [10], and different care contexts such as hospitals [11]. Despite the widespread use of PCC, there is no consensus on how it is operationalised conceptually. According to a recent scoping review [12], the most common of nine identified dimensions of person-centredness are “perception of the individual as a unique person”, “sharing of responsibility between person and practitioner”, and “formation of a therapeutic relationship". However, the literature indicates a gap between the theoretical concept and practical implementation of PCC in the hospital setting [13]. Reported barriers to provide PCC include lack of knowledge, understanding and training of healthcare professionals [5, 14], ward and institutional cultures, and inappropriate physical environment [5]. To promote PCC in hospitals, change is needed at both institutional and ward levels. Here, leadership of key personnel plays a crucial role, as staff training is not sufficient to implement PCC successfully [15–17]. At ward level, so-called change agents with clinical expertise can support staff awareness and initiate change processes in care [17]. This role of a clinical leader reflects one core competence of an Advanced Practice Nurse (APN) [18], a specialised nurse with a master’s degree, possessing expert knowledge, complex decision-making skills, and clinical competencies [19]. APN interventions for older people have shown positive outcomes in primary and long-term care. Evidence for the acute setting shows mixed results [20, 21].

As Germany is at the beginning of establishing APN, Expanded Practice Nurses (EPN) at bachelor’s level are deployed to facilitate the transition to Advanced Practice Nursing [22, 23]. Expanded nursing roles for PCC for people with cognitive impairment in hospitals are rarely described in the literature [24, 25].

The development and implementation of an EPN role is complex as various components and their interactions need to be taken into account. To comprehensively investigate complex interventions, the guidance of the British Medical Research Council (MRC) recommends the evaluation of effects, while considering underlying processes and influencing factors as well as costs [26].

In the ENROLE-acute (Expanded Nursing ROLEs for person-centred care for people with cognitive impairment in ACUTE care) project, we are developing, implementing, and evaluating a complex PCC intervention for people with cognitive impairment, delivered by EPN on peripheral wards in one acute hospital. We hypothesise that by introducing PCC, the individual needs of people with cognitive impairment will be addressed in a timely manner and thus adverse events will be reduced or avoided, leading to a reduction in the length of hospital stay.

## Objectives

This study aims to test the feasibility and clinical effectiveness of the newly developed PCC intervention. Therefore, our objectives are to assess (a) initial effects, (b) the degree and quality of implementation, mechanisms of change and contextual factors, and (c) potential cost savings of the intervention.

## Methods / Design

### Overall study design

The ENROLE-acute project follows the first and second stage of the MRC guidance [26] for complex interventions, with this study protocol addressing the feasibility stage. Protocol development adheres to the SPIRIT checklist [27] (see Additional file 1).

**Fig. 1:**
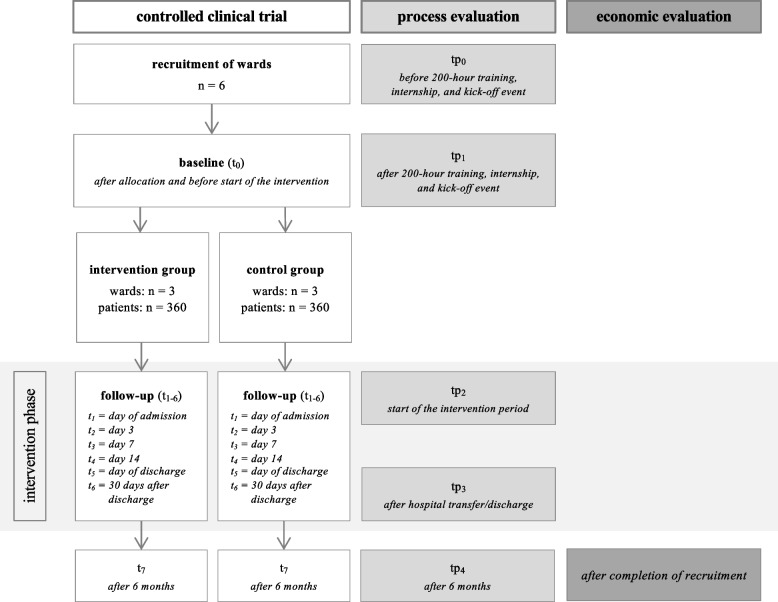
Participant timeline

To examine initial effects of the ENROLE-acute intervention, we will conduct an exploratory non-randomised controlled clinical trial (CCT), comparing the PCC intervention and optimised care as control intervention. The CCT will be accompanied by an embedded mixed-methods process evaluation to explore how and under what circumstances outcomes are achieved. In addition, we will conduct a health economic evaluation. Figure 1 summarises the overall study design.

### Setting

We will conduct the study in one university hospital in the western part of Germany. The hospital has about 1,500 beds, with inpatient care provided to 57,900 patients in 2022. According to internal hospital controlling, about 1,500 people with diagnosed cognitive impairment were treated as inpatients in the same year.

The hospital has about 12,000 employees [28]. In recent years, first EPN have been introduced. At present, there are 36 nurses working in expanded roles in wound management, oncology, psychiatry, obstetrics and gynecology, surgery, and cardiology [29, 30].

### Intervention

In the first phase of the ENROLE-acute project, we comprehensively developed the complex intervention based on two systematic reviews, two surveys [31], one qualitative interview study, and two workshops with relevant stakeholders from clinical practice, research, and representative organisations of people with cognitive impairment. We will publish details of intervention development elsewhere.

**Fig. 2:**
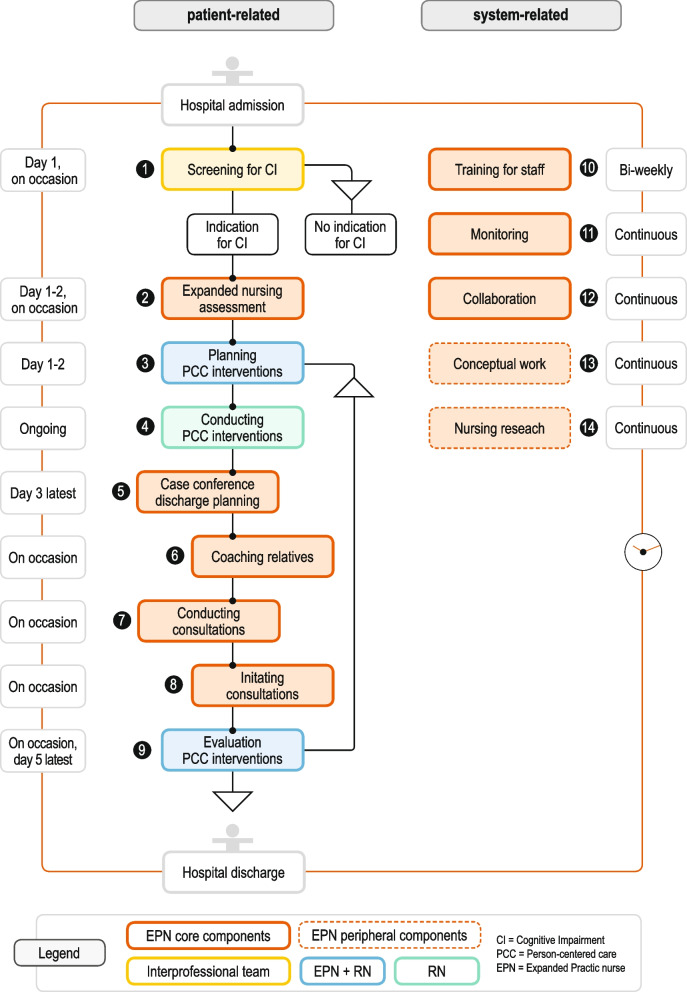
Intervention components

The complex intervention aims to foster PCC for people with cognitive impairment through EPN acting as change agents (see Fig. 2). We defined 14 components reflecting EPN’s role in collaboration with the interprofessional team. The model includes core components that are mandatory (no. 1–12) and peripheral components that are adaptable (no. 13–14) [32]. We differentiate between patient-related and system-related components. Patient-related components are aimed at individual patients and include screening and expanded nursing assessment at patient admission (no. 1–2); planning, conducting, and evaluating PCC interventions in collaboration with registered nurses (no. 3, 4 and 9); and further EPN interventions including case conferences on discharge planning, coaching relatives, and conducting and initiating consultations (no. 5–8). System-related components include EPN interventions at the organisational level, e.g., training interventions for staff, monitoring of change processes, promoting hospital-wide collaboration, conceptual work, and nursing research (no. 10–14).

Each intervention ward receives two EPN, qualified at level six of the European Qualifications Framework (bachelor’s degree or equivalent further education) [33]. EPN are integrated into the ward's work schedule and have one day each every two weeks for additional activities.

We developed different implementation strategies to prepare the field. EPN received a 200-h training regarding fundamentals of caring for people with cognitive impairment, principles of PCC, and Advanced Practice Nursing in six modules. In addition, EPN gained knowledge of established expanded nursing models in other hospitals during a 10-day internship. During the intervention period, we accompany the EPN fortnightly in two-hour coaching sessions for reflection and implementation support. We also train the interprofessional team at the beginning of the intervention phase on PCC principles and Advanced Practice Nursing during a one-hour kick-off meeting and through written information.

Team members of control wards provide optimised care with knowledge acquired through a one-time half-hour session and written information on PCC.

### Evaluation of effects

#### Participants

For details on the inclusion criteria of all participants, please see Additional file 2.

##### Patient level

We defined the following inclusion criteria for potential participants: (1) age of 65 years and older, (2) sufficient knowledge of German language, (3) hospital stay of unclear duration or over 48 h, (4) ability to consent or existence of a legal representative, and (5) present cognitive impairment (e.g., diagnosis of dementia) or risk for cognitive impairment (e.g., risk for delirium) according to predefined criteria.

We will recruit potential participants on six days a week over a time period of six months. Contact persons of the respective wards will inform eligible people with cognitive impairment and, if necessary, their representatives about the study and ask for consent to be approached by the research team for obtaining informed consent.

##### Staff level

Members of the interprofessional team are registered nurses (European Qualifications Framework level 4), nursing assistants, and physicians of all participating wards. We exclude therapists as well as service and administrative personnel as they do not form the core of the interprofessional team on a ward. We will recruit members of the interprofessional team during kick-off meetings and via superiors.

##### Cluster level

We will purposefully select six peripheral wards with a prevalence of at least 30 people with cognitive impairment per month according to data from hospital controlling. We will exclude intensive care, intermediate care, psychiatric, palliative, and paediatric wards. Based on the recommendation of nursing management, we will identify eligible wards and recruit them through individual information meetings with ward managers.

#### Outcomes, data collection and management

For an overview of primary and secondary outcomes, instruments used, data collection time points, and data sources, please see Table 1.

**Table 1:** Overview of the evaluation of effects

Variable	Instrument	Description	Source	Time points^a^	Type of variable
**PEOPLE WITH COGNITIVE IMPAIRMENT**					
Length of hospital stay	Single item	Number of days people with cognitive impairment stayed in hospital during a single admission event [34]	Patient records	t_5_	Primary outcome
Prevalence of delirium	Confusion Assessment Method – short form (CAM)	The Confusion Assessment Method consists of 4 items on (1) acute onset and/or fluctuating course, (2) inattention, (3) disorganised thinking, and (4) altered level of consciousness. Delirium is indicated sensitively if item (1) and (2), and either (3) or (4) are scored positive [35–37]	Rating by research team	t_1_, t_2_, t_3_, t_4_, t_5_	Secondary outcome
Severity of delirium	Confusion Assessment Method – Severity (CAM-S)	Delirium severity is measured using the Confusion Assessment Method by rating the items in terms of severity (0 = absent, 1 = mild, 2 = marked). The item (1) acute onset and/or fluctuating course is only rated as 0 = absent or 1 = present. Overall, the scale ranges from 0—7 points, with higher values indicating a more severe degree [38]	Rating by research team	t_1_, t_2_, t_3_, t_4_, t_5_	Secondary outcome
Delirium subtype	Delirium Motor Subtype Scale (DMSS)	The Delirium Motor Subtype Scale consists of two subscales on hyperactive (4 items) and hypoactive (7 items) subtype, classifying four different motor subtypes: hypoactive, hyperactive, mixed or no motor subtype. Each item is scored dichotomously (present or absent) over the past 24 h. Hyperactive delirium is positive, when two items on the hyperactive subscale are present. Hypoactive delirium is positive, when either “decreased amount of activity” or “decreased speed of actions” in combination with at least one other item is present. Mixed type is positive, when both hyper- and hypoactive delirium was positive within the last 24 h [39, 40]	Rating by registered nurses	t_1_, t_2_, t_3_, t_4_, t_5_	Secondary outcome
Prevalence of pain	Numeric Rating Scale (NRS)	The Numeric Rating Scale is a 11-point scale ranging from 0 (no pain) to 10 (worst possible pain) [41]. A value ≥ 1 indicates pain	Self-rating by people with cognitive impairment	t_1_, t_2_, t_3_, t_4_, t_5_	Secondary outcome
	Pain Assessment in Advanced Dementia (PAINAD-G)	If self-assessment is not possible with NRS, the Pain Assessment in Advanced Dementia (PAINAD-G) will be used. PAINAD-G consists of five items (breathing, negative vocalisation, facial expressions, body language, consolability) resulting in a score from 0 (no pain) to 10 (worst possible pain) [42, 43]. A score > 2 is defined as an indicator for pain [44]	Rating by research team	t_1_, t_2_, t_3_, t_4_, t_5_	Secondary outcome
Prevalence of unrecognised pain	NRS / PAINAD-G	Unrecognised pain is defined by identified pain by the research team and, at the same time, no documentation of pain or administration of medication on demand in patient records	Discrepancy between rating by research team and patient records	t_1_, t_2_, t_3_, t_4_, t_5_	Secondary outcome
Prevalence of agitation	Cohen Mansfield Agitation Inventory (CMAI, hospital version)	The hospital version of the Cohen Mansfield Agitation Inventory consists of 9 items on a 7-point scale measuring frequency of agitated behaviour (including two subscales on aggressive and non-aggressive behaviour) with a time reference of one week. A score between 9 and 63 can be achieved, with higher scores reflecting a higher level of agitation [45, 46]	Retrospective rating by registered nurses	t_3_, t_4_, t_5_	Secondary outcome
Anxiety, stress, and depression	Depression Anxiety Stress Scales (DASS) – short form	The Depression Anxiety Stress Scales consist of 21 items, which are divided into 7 items each for the constructs depression, anxiety and stress. On a 4-point scale, the extent is rated from 0 = not at all true to 4 = very true or true most of the time. The time reference is a period of one week. A sum score is calculated for each subscale, with higher values indicating a higher degree of the negative emotional state. A score of 10 for depression and stress, as well as a score of 6 for anxiety can be assumed to indicate an increased expression of these characteristics [47]	Self-rating by people with cognitive impairment	t_1,_ t_3_, t_4_, t_5_	Secondary outcome
Quality of sleep	Richards-Campbell-Sleep-Questionnaire (RCSQ)	The Richards-Campbell-Sleep-Questionnaire consists of 5 items on sleep depth, falling asleep, being awake, falling asleep again and sleep quality. The evaluation of the individual items refers to the previous night and is done using a visual analogue scale, with 0 mm representing the worst sleep and 100 mm the best sleep [48, 49]	Self-rating by people with cognitive impairment	t_2_, t_3_, t_4_, t_5_	Secondary outcome
	Outcome-oriented nursing assessment AcuteCare (epa-AC ©^b^)	Sleep problems are described in epa-AC using two items, the presence of falling asleep and staying asleep as well as a disturbed sleep–wake rhythm [50]	Patient records	t_2_, t_3_, t_4_, t_5_	Secondary outcome
Quality of life	Bath Assessment of Subjective Quality of Life in Dementia (BASQID)	The Bath Assessment of Subjective Quality of Life in Dementia includes 14 items divided into two subscales on life satisfaction and feeling of a positive quality of life. Both subscales are used with a 5-point Likert scale ranging from 0 = not at all satisfied or not at all to 4 = extremely satisfied or very satisfied. Overall, the scale results in a transformed score of 0—100 points, with higher values indicating a higher quality of life. Three additional questions provide a comprehensive assessment of quality of life, health, and memory performance and are evaluated separately [51, 52]	Self-rating by people with cognitive impairment	t_5_	Secondary outcome
	QUALIDEM	The QUALIDEM uses 37 items to measure the quality of life of people with dementia, with a selection of 18 items being used for people with severe dementia. The 18-item version addresses the domains care relationship, positive affect, negative affect, restless and tense behaviour, social relationships, and social isolation. Responses are made using a 7-point Likert scale that asks for frequency. Although an analysis within domains is recommended, a total score of 0—168 points can also be calculated, with higher values representing a higher quality of life [53, 54]	Retrospective rating by registered nurses	t_5_	Secondary outcome
Person-centred care	Individualised Care Scale (ICS)	The Individualised Care Scale consists of 17 items measuring the perspective of people with cognitive impairment on implementation of individualised care during hospitalisation. Items are rated with a 5-point Likert scale ranging from 1 = strongly disagree to 5 = strongly agree. A general score is calculated as mean value between 1 and 5, with higher values indicating more individualised care [55, 56]	Self-rating by people with cognitive impairment	t_5_	Secondary outcome
Falls	Single items	Number of falls documented	Patient records	t_5_	Secondary outcome (safety outcome)
Physical restraints	Single items	Occurrence of the use of physical restraints	Retrospective rating by registered nurses	t_2_, t_3_, t_4_, t_5_	Secondary outcome (safety outcome)
Prescription of antipsychotics	Predefined list	Newly prescribed antipsychotic medication during hospitalisation	Patient records	t_5_	Secondary outcome (safety outcome)
Mortality	Single items	People with cognitive impairment that died during data collection	Patient records	t_6_	Secondary outcome (safety outcome)
Stability of care arrangements	Single items	• Number of readmissions within 30 days • Perceived stability of care arrangement	Telephone interview with people with cognitive impairment and/or their relatives	t_6_	Secondary outcome (safety outcome)
Sociodemographic variables	Single items	• Age in years • Gender: Male, female, or divers • Care dependency levels ranging from 1 to 5 • Care situation before admission	Patient records	t_1_	Control variable
Admission diagnosis	Single items	Admission diagnosis as documented	Patient records	t_5_	Control variable
Type of admission	Single items	Emergency or planned admission	Patient records	t_1_	Control variable
Previous hospital admissions	Single items	Number of hospital admissions within the last seven days	People with cognitive impairment and/or their relatives	t_1_	Control variable
Admission diagnosis-related (diagnosis related groups) length of hospital stay	Single items	Mean stated length of stay of the patient's diagnosis-related group	Patient records	t_5_	Control variable
Or Ward-related length of hospital stay		Minimum length of stay of special wards, e.g., based on rehabilitation programmes	Ward managers	t_0_	
Type of discharge	Single items	Discharge home, transfer	Patient records	t_5_	Control variable
Self-care ability	Self-care Ability Index (epa-AC ©^b^)	The Self-care Ability Index consists of 10 items: mobility, grooming and dressing (lower and upper body), eating and drinking, excretion, and cognition / consciousness. The items are rated every day on a 4-point Likert scale, with high scores reflecting a high self-care capacity. The scale ranges from 10 points (= impaired self-care ability) to 40 points (= full self-care ability) [50]	Patient records	t_1_	Control variable
Cognition	Montreal Cognitive Assessment (MoCA)	The Montreal Cognitive Assessment consists of 12 tasks on short-term memory recall, visuospatial abilities, executive functions, attention, concentration, working memory, language, and orientation. The maximum score achievable is 30 points. Scores between 28—25 indicate mild cognitive impairment, scores between 10—17 indicate moderate cognitive impairment, and scores < 10 indicate severe cognitive impairment [57]	Rating by research team	t_1_	Control variable
Comorbid disease status	Charlson Comorbidity Index (CCI)	The Charlson Comorbidity Index consists of 19 clinical conditions, each of which is weighted to produce a sum score. Higher scores indicate a greater risk of mortality and more severe comorbid conditions [58, 59]	Patient records	t_5_	Control variable
**STAFF**					
Distress associated with difficult behaviour for people with cognitive impairment	Residents’ Challenging Behaviour related Distress Index	The Residents’ Challenging Behaviour related Distress Index includes 10 items that address altered behaviour such as hallucinations, instability, anxiety, depression, apathy, euphoria, limited communication skills, disinhibition, aggression, and impaired motor skills. All items are rated in terms of the degree of distress 1 = does not stress me at all, 2 = stresses me somewhat, and 3 = stresses me a lot. The score ranges from 0—100 points, with a higher score indicating higher distress [60]	Self-rating by staff	t_0_, t_7_	Secondary outcome
Person-centred care	Person-Centred Practice Inventory – Staff (PCPI-S)	The Person-Centred Practice Inventory—Staff includes 59 items covering 17 different constructs of person-centredness with respect to 3 domains: prerequisites, care environment, and person-centred processes. The items are measured on a 5-point Likert scale from 0 = strongly disagree to 4 = strongly agree. Overall, the scale shows a sum score of 0—100, with higher scores representing greater agreement with person-centredness [61, 62]	Self-rating by staff	t_0_, t_7_	Secondary outcome
Sociodemografic variables	Single items	• Age in years • Gender	Self-rating by staff	t_0_, t_7_	Secondary outcome

^a^t_0_ = before implementation of intervention; t_1_ = admission; t_2_ = day 3 of hospital stay; t_3_ = day 7 of hospital stay; t_4_ = day 14 of hospital stay; t_5_ = discharge from the ward; t_6_ = 30 days after discharge; t_7_ = 6 months after baseline

^b^With Outcome-oriented nursing assessment AcuteCare (epa-AC ©) the patient's abilities and impairments are assessed daily by registered nurses using the four-level epa point system and recorded in electronic form [50]

##### Patient level

At patient level, we will collect data at a maximum of six timepoints: t_1_ (at admission), t_2_ (at day 3), t_3_ (at day 7), t_4_ (at day 14), t_5_ (at discharge), and t_6_ (30 days after discharge). We will collect study documentation data (e.g., participant flow) paper-based and outcome data electronically using REDCap®. Trained study staff will accompany people with cognitive impairment for self-rating. Proxy-rating will be carried out by trained study staff or nurses who have been responsible for the participant for at least one shift within the past 24 h. In case of non-participation of people with cognitive impairment in the study, we will document the reason.

Data at patient level will be documented using participant codes. We will keep identification lists and paper-based documents under lock, separate from other study material and only accessible to data collectors. Electronic data will be password-protected and only accessible to the research team. We will automatically carry out a complete backup of the data every day. With the set-up of the database, we have stored definitions (e.g., minimum and maximum) for each value to be entered, which minimises the entry of invalid data. In addition, the biostatistician will perform quality and plausibility checks (data validation).

##### Staff level

For outcomes at staff level, we will use a paper-based questionnaire at two timepoints: t_0_ (before intervention phase) and t_7_ (after six months). We will collect the data pseudonymously and use a self-generated code to allow both sets of data of one participant to be matched over the course of the study. To increase retention, we will use incentives and email reminder to ward managers.

Only data collectors will have access to the paper-based questionnaires, which are kept under lock. We will keep potentially identifying data separate from the rest of the study material. One person will conduct electronic data entry and random samples of the record will be double-checked by another person. In case of discrepancies, the entire data entry will be double-checked.

#### Assignment of intervention

Allocation of wards as intervention or control ward was based on ward managers’ decision to participate in the intervention. Participants’ assignment will be determined by their wards’ assignment.

#### Blinding

Due to the nature of the intervention, blinding of participants, EPN providing the intervention, and researchers collecting data is not feasible. However, we will blind the biostatistician during data analyses regarding group allocation of clusters (neutral group IDs) and participating people with cognitive impairment.

#### Sample size

The sample size is based on a recent retrospective cohort study which investigates the length of hospital stay in older people living with dementia by matching people without dementia in Germany [63]. We assume a mean reduction in the length of hospital stay of three days (intervention vs. control). With a standard deviation of 18 vs. 16 days, an intraclass correlation coefficient of 0.05 [64], and three clusters per treatment group with 120 people with cognitive impairment each, the adjusted two-sample t-test will reach a power of 81.5% at one-sided significance level of 50% [65]. Thus, this exploratory trial will be sufficiently powered to show the hypothesized direction of effect only.

#### Data analysis

Statistical analysis is according to intention-to-treat (no exclusions). We will evaluate the primary outcome length of hospital stay by the stratified log-rank test (death is censored). Nota bene, the stratified log-rank test has very similar characteristics as the stratified van-Elteren rank-sum test and is available in many (statistical) software packages. We will use a propensity score approach (PSA) to balance individual characteristics between participants in intervention and control groups. In the multivariable logistic regression for PSA, we will use the independent variables age, gender, severity of cognitive impairment, Charlson Comorbidity Index, admission type, expected admission diagnosis-related or ward-related length of stay, and hospitalisations during the last 7 days [66, 67] as these variables are likely to be associated with outcome. Moreover, we will carefully monitor mortality in both arms, although we do not expect any difference. We will evaluate secondary outcome measures by (generalised) mixed models for repeated measures over time. If feasible, we will take outcome measures (particularly those reported by people with cognitive impairment) over the course of hospital stay. Thus, we may need to evaluate the outcome trajectories by random coefficient models. We will categorise and compare safety outcomes by frequency, relatedness, seriousness, and severity. Moreover, we will summarise variables by mean, standard deviation, and percentiles (0, 25, 50, 75, and 100) respectively by absolute and relative frequencies (percentage). Due to the small number of clusters, we will at least account for them in a fixed effect analysis. Though we will attempt to estimate corresponding random effects (e.g., to derive intraclass correlation), this seems prone to bias.

### Evaluation of processes

The process evaluation is based on a convergent parallel mixed-methods approach [68]. Theoretical foundations are the MRC guidance for process evaluations of complex interventions [69] and Grant’s framework on process evaluation for cluster-randomised trials [70]. For the design and conduct of the study, we developed a logic model that illustrates expected causal pathways of the ENROLE-acute intervention and potential moderating effects of relevant contextual factors (the logic model will be published as part of the intervention development). We used the logic model’s domains (implementation, mechanisms of change, and context) to organise all process variables in a predefined analysis-plan. Our purpose is that by evaluating processes before, during, and after the intervention period, observed effects can be interpreted in the light of how the complex intervention actually works. For an overview of domains, questions, data collection, participants, and time points, please see Table 2.

**Table 2:** Overview of the evaluation of processes

**Domains**		**Questions**	**Data collection**	**Participants**	**Time points**^a^
Implementation	Recruitment of expanded practice nurses	How is recruitment done?	Documentation of recruitment	–	tp_0_
		Who is (not / no longer) participating?	Documentation of recruitment and retention	–	tp_0_, occasion-related
		Why do (potential) expanded practice nurses (not / no longer) participate?	Documentation of recruitment and retention	–	tp_0_, occasion-related
			Qualitative interviews on motivation and reasons for (non-)participation	(Potential) expanded practice nurses	Occasion-related
	Delivery to expanded practice nurses	What is delivered to the expanded practice nurses? Is it what the researchers intend?	Documentation of contact hours in training, internship, and coaching sessions	–	tp_1_, ongoing
			Review of training and coaching material and internship report	–	tp_1_, ongoing
			Review of coaching session protocols	–	tp_4_
	Recruitment of hospital wards	How is recruitment done?	Documentation of recruitment	–	tp_0_
		Who is (not / no longer) participating?	Documentation of recruitment and retention	–	tp_0_, occasion-related
		Why do wards (not / no longer) participate?	Documentation of recruitment and retention	–	tp_0_, occasion-related
			Qualitative interviews on motivation and reasons for (non-)participation	Ward managers	Occasion-related
	Delivery to interprofessional team members on hospital wards	What is delivered to the interprofessional team members? Is it what the researchers intend?	Documentation of participation in the kick-off event	–	tp_1_
			Review of the kick-off event and written information material	–	tp_1_
			Documentation of the expanded practice nurses’ activities in the interprofessional team in a logbook	Expanded practice nurses	Ongoing
			Review of coaching session protocols	–	tp_4_
	Recruitment of people with cognitive impairment and their relatives	How is recruitment done?	Documentation of recruitment	–	Ongoing
		Who is (not / no longer) participating?	Documentation of recruitment and retention	–	Ongoing
		Why do people with cognitive impairment and their relatives (not / no longer) participate?	Documentation of recruitment and retention	–	Ongoing
	Delivery to people with cognitive impairment and their relatives	What is delivered to the people with cognitive impairment and their relatives? Who is receiving? Is it what the researchers intend?	Documentation of the expanded practice nurses’ activities with people with cognitive impairment and their relatives in a logbook	Expanded practice nurses	Ongoing
			Review of coaching session protocols	–	tp_4_
			Qualitative dyad interviews on expanded practice nurses’ and interprofessional team members’ activities	People with cognitive impairment and their relatives	tp_3_
			Focus groups on expanded practice nurses’ and interprofessional team members’ activities	Expanded practice nurses, members of the interprofessional team	tp_4_
Mechanisms of change	Response of expanded practice nurses	Based on the Kirkpatrick model:	Reflection forms on satisfaction with and perceived usefulness of training, internship, and coaching sessions (1)	Expanded practice nurses	tp_1_
		How do expanded practice nurses react? (1) To what extent is there learning success? (2) To what extent can the learning be transferred to behaviour in everyday care? (3) To what extent are there results? (4)			
			Non-moderated, guided focus group on satisfaction with and perceived usefulness of training, internship, and coaching sessions as well as expectations for the intervention period (1, 3)	Expanded practice nurses	tp_1_
			Questionnaire on self-assessed knowledge (2)	Expanded practice nurses	tp_0_, tp_1_
			Documentation of the expanded practice nurses’ activities in a logbook (3)	Expanded practice nurses	Ongoing
			Review of coaching session protocols (3)	–	tp_4_
			Qualitative interviews on self-perceived skills and competences in core tasks (3, 4)	Expanded practice nurses	tp_4_
			Questionnaire on role understanding and collaboration (4)	Expanded practice nurses	tp_0_, tp_1_, tp_4_
	Response of interprofessional team members on hospital wards	How do interprofessional team members react? (1) To what extent do interprofessional team members accept what is delivered? (2) To what extent are there results? (3)	Reflection forms on satisfaction with and perceived usefulness of the kick-off event and written information material (1, 2)	Interprofessional team members	tp_1_, tp_4_
			Questionnaire on acceptability and role understanding of as well as collaboration with expanded practice nurses (1–3)	Interprofessional team members	tp_0_, tp_4_
			Questionnaire on attitudes towards and satisfaction with (person-centred) care for people with cognitive impairment (3)	Interprofessional team members	tp_0_, tp_4_
	Response of people with cognitive impairment and their relatives	How do people with cognitive impairment and their relatives react? (1) To what extent do people with cognitive impairment and their relatives accept what is delivered? (2) To what extent are there results? (3)	Qualitative dyad interviews on acceptability and role understanding of expanded practice nurses as well as satisfaction with expanded practice nurses’ and interprofessional team members’ activities (1–3)	People with cognitive impairment and their relatives	tp_3_
			Focus groups on patients’ acceptability and role understanding of expanded practice nurses as well as satisfaction with expanded practice nurses’ and interprofessional team members’ activities (1–3)	Expanded practice nurses, members of the interprofessional team	tp_4_
	Maintenance	To what extent are the processes maintained over time?	Documentation of contact hours in coaching sessions	–	Ongoing
			Documentation of the expanded practice nurses’ activities in the interprofessional team and with people with cognitive impairment and their relatives in a logbook	Expanded practice nurses	Ongoing
			Review of coaching session protocols	–	tp_4_
			Focus groups on expanded practice nurses’ and interprofessional team members’ activities	Expanded practice nurses, members of the interprofessional team	tp_4_
		What are the reasons for (lack of) maintenance?	Focus groups on staff intention and reasons for (lack of) expanded practice nurses’ and interprofessional team members’ activities	Expanded practice nurses, members of the interprofessional team	tp_4_
			Qualitative interviews on organisational intention and reasons for (lack of) expanded practice nurses’ and interprofessional team members’ activities	Ward managers	tp_4_
	Unintended consequences	Do unintended changes occur? What are the reasons for unintended changes?	Focus groups on unintended consequences of training, internship, and coaching sessions, kick-off event and written information material or expanded practice nurses’ and interprofessional team members’ activities	Expanded practice nurses, members of the interprofessional team	tp_4_
			Qualitative interviews on unintended consequences of training, internship, and coaching sessions, kick-off event and written information material or expanded practice nurses’ and interprofessional team members’ activities	Ward managers	tp_4_
			Qualitative dyad interviews on unintended consequences of expanded practice nurses’ and interprofessional team members’ activities	People with cognitive impairment and their relatives	tp_3_
Context	Micro level	What are the characteristics of the expanded practice nurses?	Questionnaire on sociodemographic, personal, and professional characteristics that may influence the intervention	Expanded practice nurses	tp_0_
		What are the characteristics of the interprofessional team members and ward managers?	Questionnaire on sociodemographic, personal, and professional characteristics that may influence the intervention	Members of the interprofessional team, ward managers	tp_2_
		What are the characteristics of the people with cognitive impairment and their relatives?	Patient records	–	tp_3_
			Qualitative dyad interviews on personal characteristics that may influence the intervention	People with cognitive impairment and their relatives	tp_3_
		To what extent do the characteristics act as barriers or facilitators?	Qualitative interviews on perceived barriers and facilitators	Expanded practice nurses, ward managers, nursing development unit	tp_4_
			Questionnaire on perceived barriers and facilitators	Members of the interprofessional team	tp_4_
			Qualitative dyad interviews on perceived barriers and facilitators	People with cognitive impairment and their relatives	tp_3_
	Meso level	What are the characteristics of the wards?	Questionnaire on ward characteristics that may influence the intervention	Ward managers	tp_4_
		What are the characteristics of the University Hospital Cologne?	Qualitative interviews on hospital characteristics that may influence the intervention	Nursing management, nursing development unit	tp_4_
		To what extent do the characteristics act as barriers or facilitators?	Qualitative interviews on perceived barriers and facilitators	Expanded practice nurses, ward managers, nursing management, nursing development unit	tp_4_
			Questionnaire on perceived barriers and facilitators	Members of the interprofessional team	tp_4_
	Macro level	What are the characteristics of the society?	Qualitative interviews on society characteristics that may influence the intervention	Nursing management, nursing development unit	tp_4_
		To what extent do the characteristics act as barriers or facilitators?	Qualitative interviews on perceived barriers and facilitators	Expanded practice nurses, nursing management, nursing development unit	tp_4_

^a^tp_0_ = before training, internship, and kick-off event; tp_1_ = after training, internship, and kick-off event; tp_2_ = start of the intervention period; tp_3_ = after transfer/discharge of people with cognitive impairment; tp_4_ = 6 months after the start of the intervention period

#### Participants

We will ask members of the three hospital wards in the ENROLE-acute intervention group to participate in the process evaluation. Target groups of the quantitative and qualitative parts are all EPN, members of the interprofessional team, and ward managers. The qualitative part additionally includes people with cognitive impairment and their relatives as well as members of the hospital’s nursing management and nursing development unit. For the eligibility criteria for each target group, please see Additional file 2.

When selecting participants, we will combine different sampling strategies. For all quantitative parts and the qualitative parts on EPN level, we strive to recruit all members of the relevant target groups. For the remaining explorations, we will form subsamples through purposeful sampling. A predefined sampling plan ensures that different forms of cognitive impairment respectively health professions as well as all three clusters are represented in the sample. The targeted sample size will be guided by reaching data saturation.

For recruitment, we will invite members of the target groups both orally and in writing to participate in the study.

#### Data collection and management

We will collect both qualitative and quantitative process data on cluster and individual level at five time points: tp_0_ (before training, internship, and kick-off event), tp_1_ (after training, internship, and kick-off event), tp_2_ (start of the intervention period), tp_3_ (after transfer or discharge of people with cognitive impairment), and tp_4_ (6 months after start of the intervention period).

The qualitative part of the process evaluation comprises individual (EPN, ward managers, members of the nursing management and the nursing development unit) and dyad interviews (people with cognitive impairment and their relatives) as well as moderated and non-moderated focus groups (EPN, members of the interprofessional team). We will collect data face-to-face, by phone or by web-based video conferencing. Nursing researchers trained in qualitative methodology will guide the data collections. We will audiotape and transcribe interviews and group discussions verbatim.

To collect quantitative data, we will use paper-based questionnaires (EPN, members of the interprofessional team, ward managers) and reflection forms (EPN, members of the interprofessional team). A self-generated identification code will allow us to link participants’ data over time. To increase retention, we will use incentives and email reminder. After data entry, we will perform quality and plausibility checks.

For collecting quantitative and qualitative data, we refer to pre-existing and approved instruments. Only if this is not possible, we will use self-developed instruments. We conduct pretests of all instruments to be used.

In addition, we will gather different process documents and materials (e. g., structured notes on recruitment and retention, protocols and attendance lists, teaching and learning materials, and EPN logbooks).

We will store data both pseudonymously (EPN and ward managers) and anonymously (people with cognitive impairment and their relatives, interprofessional team members, and members of the nursing management and the nursing development unit), while documenting the time point and cluster assignment on data sheets. Data protection follows current data protection laws. We will keep paper-based documents under lock. Electronic data will be password-protected. We will store potentially identifying data separate from the rest of the study material. Data will only be accessible to the research team.

#### Data analysis

For analysing the process data, we will use a stepwise approach. First, we will analyse qualitative and quantitative data separately. Subsequently, we will merge data during the interpretation of the results.

For analysing the qualitative data (interviews and focus groups), we will conduct a thematic framework analysis [71] with a deductive-inductive approach based on the logic model. Furthermore, we will use a qualitative content analysis [72] applying a deductive-inductive approach to review the structured process documents and materials as well as open-ended questions in the questionnaires. We will conduct all qualitative analyses in the software MAXQDA [73]. To promote credibility, two persons will analyse data and discuss findings within the research team.

We will analyse quantitative data (questionnaires and reflection forms) by means of descriptive statistics (frequency, distributional location, and spread) using the software IBM SPSS Statistics [74].

We will combine qualitative and quantitative findings regarding key process variables and outcomes incorporated in our logic model.

### Economic evaluation

We will perform a cost-consequence analysis from the hospital perspective [75].

#### Data collection and management

We will use claims data from the hospital to determine the potential cost savings of the intervention. These will be available at the hospital controlling department. This data includes Diagnosis Related Groups (DRG) charges, length of hospital stay, cost weight, gender, and primary and secondary International Statistical Classification of Diseases and Related Health Problems (ICD) diagnoses. The data will be delivered pseudonymously to the evaluators via a secure data exchange platform. In addition, we will collect training costs consisting of salaries of the nursing staff, the costs of the lecturers as well as material costs for premises, catering and travel costs.

#### Data analysis

In the cost-consequence analysis, we will compare the intervention group with the control group in terms of total inpatient costs, length of hospital stay, and cost weight. We will add the intervention costs (e.g., training costs) to the DRG charges of the intervention group. We will analyse differences (possibly after log-transformation) between the mean total costs using a t-test. In addition, we will perform additional analyses according to gender, age groups, and cost weight using t-test, Mann–Whitney-U-test, and other statistical tests. The analysis will be performed with IBM SPSS [74] and Microsoft Access.

## Dissemination

We will publish main study results in international, peer reviewed journals. In addition, we will present our results at relevant scientific conferences and meetings. We will report the results based on this study protocol as well as the recommendations of current reporting guidelines. Main study information (e.g., intervention material and results) will be freely available at our project website (https://www.enrole-acute.uni-koeln.de/).

We will share authorship between persons involved in the study following the current guidelines of the International Committee of Medical Journal Editors. Persons not directly involved in the study will not be granted authorship.

## Discussion

Complex interventions are considered most promising in promoting PCC [76]. The ENROLE-acute intervention builds on implementing an expanded nursing role as a core element to enhance PCC for people with cognitive impairment in acute care. Intervention development and testing are theory-based and follow recent guidance [26, 69]. The planned exploratory CCT will provide insights into the intervention’s initial effects, with the primary outcome focusing on patient length of hospital stay. Since there is a lack of high-quality studies to evaluate PCC interventions for people with cognitive impairment using expanded nursing roles in acute care, our study will provide important evidence. With the accompanying mixed-methods process evaluation, we will additionally examine the intervention’s implementation, mechanisms of change, and contextual factors. Analysing these underlying processes will lead to more transparency in trial results and a comprehensive understanding of how and under what circumstances the intervention will actually work. The additional economic analysis will increase our knowledge regarding the costs of the intervention.

However, some limitations of the study must be considered. Due to practical reasons, randomisation of the clusters will not be feasible. This may lead to differences between the groups at baseline resulting in a high risk of selection bias. However, we will consider these differences in the statistical analyses. Due to the nature of the intervention, blinding of participants and researchers during the study period will also not be possible. The knowledge of the group allocation might affect both the participants’ answers and data collection by researchers. At least the statistician will be blinded during data analysis. Finally, staff turnover during the six-month intervention period might result in differences between staff participating in the data collection at baseline and follow-up.

Despite the limitations, the study results will support the future development and implementation of new as well as the optimisation of existing PCC interventions based on expanded nursing roles. Our implications can particularly inform evidence-based decision making at the management and policy level. Additionally, our results will provide valuable information for planning and conducting large-scale trials as claimed in the third and fourth stage of the MRC guidance [26].

### Supplementary Information

**Additional file 1.** SPIRIT checklist.

**Additional file 2.** Eligibility criteria.

## Data Availability

Some of the datasets generated and analysed during the current study will be available from the last author upon reasonable request.

## Abbreviations

APN: Advanced Practice Nurse
CCT: Controlled clinical trial
ENROLE-acute: Expanded Nursing ROLEs for person-centred care for people with cognitive impairment in ACUTE care
EPN: Expanded Practice Nurse
DRG: Diagnosis Related Groups
ICD: International Statistical Classification of Diseases and Related Health Problems
MRC: British Medical Research Council
PCC: Person-centred care
PSA: Propensity score approach

## References

[1] Bundesamt für Statistik: Eckdaten der Krankenhauspatientinnen und -patienten. https://www.destatis.de/DE/Themen/Gesellschaft-Umwelt/Gesundheit/Krankenhaeuser/Tabellen/entlassene-patienten-eckdaten.html (2021). Accessed 07 Nov 2023.

[2] Kracht F, Boekholt M, Schumacher-Schönert F, Nikelski A, Chikhradze N, Lücker P (2021). Describing people with cognitive impairment and their complex treatment needs during routine care in the hospital – cross-sectional results of the intersec-CM study. BMC Geriatr.

[3] Bickel H, Hendlmeier I, Heßler JB, Junge MN, Leonhardt-Achilles S, Weber J (2018). The prevalence of dementia and cognitive impairment in hospitals. Dtsch Arztebl Int.

[4] Fogg C, Griffiths P, Meredith P, Bridges J (2018). Hospital outcomes of older people with cognitive impairment: an integrative review. Int J Geriatr Psychiatry.

[5] Gwernan-Jones R, Abbott R, Lourida I, Rogers M, Green C, Ball S (2020). The experiences of hospital staff who provide care for people living with dementia: a systematic review and synthesis of qualitative studies. Int J Older People Nurs.

[6] National Institute for Health and Care Excellence. Dementia: assessment, management and support for people living with dementia and their carers. 2018. https://www.nice.org.uk/guidance/ng97/resources/dementia-assessment-management-and-support-for-people-living-with-dementia-and-their-carers-pdf-1837760199109 . Accessed 7 Nov 2023. 30011160

[7] Deutsches Netzwerk für Qualitätsentwicklung in der Pflege. Expertenstandard Beziehungsgestaltung in der Pflege von Menschen mit Demenz. 2019. https://www.dnqp.de/expertenstandards-und-auditinstrumente/#c4624162. Accessed 10 Dec 2023.

[8] Fazio S, Pace D, Maslow K, Zimmerman S, Kallmyer B (2018). Alzheimer’s association dementia care practice recommendations. Gerontologist.

[9] Kitwood T (1997). Dementia reconsidered: The person comes first.

[10] McCormack B, McCance T (2016). Person-centred practice in nursing and health care: theory and practice.

[11] Janerka C, Leslie GD, Gill FJ (2023). Development of patient-centred care in acute hospital settings: a meta-narrative review. Int J Nurs Stud.

[12] Sturgiss EA, Peart A, Richard L, Ball L, Hunik L, Chai TL (2022). Who is at the centre of what? A scoping review of the conceptualisation of ‘centredness’ in healthcare. BMJ Open.

[13] Turner A, Eccles FJR, Elvish R, Simpson J, Keady J (2017). The experience of caring for patients with dementia within a general hospital setting: a meta-synthesis of the qualitative literature. Aging Ment Health.

[14] Røsvik J, Rokstad AMM (2020). What are the needs of people with dementia in acute hospital settings, and what interventions are made to meet these needs? A systematic integrative review of the literature. BMC Health Serv Res.

[15] Santana MJ, Manalili K, Jolley RJ, Zelinsky S, Quan H, Lu M (2018). How to practice person-centred care: a conceptual framework. Health Expect.

[16] Sanatinia R, Crawford MJ, Quirk A, Hood C, Gordon F, Crome P (2020). Health services and delivery research. identifying features associated with higher-quality hospital care and shorter length of admission for people with dementia: a mixed-methods study.

[17] Handley M, Bunn F, Goodman C (2017). Dementia-friendly interventions to improve the care of people living with dementia admitted to hospitals: a realist review. BMJ Open.

[18] Tracy MF, O'Grady ET, Phillips SJ (2022). Advanced practice nursing: an integrative approach.

[19] ICN. Guidelines on Advanced Practice Nursing. International Council of Nurses. 2020. https://www.icn.ch/system/files/documents/2020-04/ICN_APN%20Report_EN_WEB.pdf. Accessed 07 Nov 2023.

[20] Morilla-Herrera JC, Garcia-Mayor S, Martín-Santos FJ, Kaknani Uttumchandani S, Leon Campos Á, Caro Bautista J (2016). A systematic review of the effectiveness and roles of advanced practice nursing in older people. Int J Nurs Stud.

[21] Chavez KS, Dwyer AA, Ramelet A-S (2018). International practice settings, interventions and outcomes of nurse practitioners in geriatric care: a scoping review. Int J Nurs Stud.

[22] Beil-Hildebrand MB, Smith HB (2022). Comparative analysis of advanced practice nursing: contextual and historical influences in North American and German-Speaking European Countries. Policy Polit Nurs Pract.

[23] Maier CB, Aiken LH, Busse R. Nurses in advanced roles in primary care: Policy levers for implementation. 2017. https://www.oecd-ilibrary.org/social-issues-migration-health/nurses-in-advanced-roles-in-primary-care_a8756593-en. Accessed 07 Nov 2023.

[24] Schindel Martin L, Gillies L, Coker E, Pizzacalla A, Montemuro M, Suva G (2016). An education intervention to enhance staff self-efficacy to provide dementia care in an acute care hospital in Canada. Am J Alzheimers Dis Other Demen.

[25] Yevchak A, Fick DM, Kolanowski AM, McDowell J, Monroe T, LeViere A (2017). Implementing nurse-facilitated person-centered care approaches for patients with delirium superimposed on dementia in the acute care setting. J Gerontol Nurs.

[26] Skivington K, Matthews L, Simpson SA, Craig P, Baird J, Blazeby JM (2021). A new framework for developing and evaluating complex interventions: update of medical research council guidance. BMJ.

[27] Chan AW, Tetzlaff JM, Altman DG, Laupacis A, Gøtzsche PC, Krleža-Jerić K (2013). SPIRIT 2013 statement: defining standard protocol items for clinical trials. Ann Intern Med.

[28] Uniklinik Köln. Jahresabschluss 2021. https://webstatic.uk-koeln.de/im/dwn/pboxx-pixelboxx-246336/jahresabschluss-2021.pdf. Accessed 07 Nov 2023.

[29] Uniklinik Köln: Pflege. https://www.uk-koeln.de/patienten-besucher/pflege/ (2023a). Accessed 07 Nov 2023.

[30] Uniklinik Köln: Pflegerische Experten. https://www.uk-koeln.de/patienten-besucher/pflege/pflegerische-experten/ (2023b). Accessed 07 Nov 2023.

[31] von der Lühe V, Roos M, Adams A, Scholten N, Köpke S, Dichter MN. Evolution of advanced practice nursing in acute care in Germany: a cross-sectional study of nurses’ scope of practice. Int Nurs Rev. 2023;0:0. 10.1111/inr.12907 37965870

[32] Damschroder LJ, Aron DC, Keith RE, Kirsh SR, Alexander JA, Lowery JC (2009). Fostering implementation of health services research findings into practice: a consolidated framework for advancing implementation science. Implement Sci.

[33] European Union. Description of the eight EQF levels. https://europa.eu/europass/en/description-eight-eqf-levels. Accessed 07 Nov 2023.

[34] Huntley DA, Cho DW, Christman J, Csernansky JG (1998). Predicting length of stay in an acute psychiatric hospital. Psychiatr Serv.

[35] Inouye SK, van Dyck CH, Alessi CA, Balkin S, Siegal AP, Horwitz RI (1990). Clarifying confusion: the confusion assessment method. A new method for detection of delirium. Ann Intern Med..

[36] Hestermann U, Backenstrass M, Gekle I, Hack M, Mundt C, Oster P (2009). Validation of a German version of the confusion assessment method for delirium detection in a sample of acute geriatric patients with a high prevalence of dementia. Psychopathology.

[37] Inouye SK. The Confusion Assessment Method (CAM): Training Manual and Coding Guide. 2003. https://americandeliriumsociety.org/wp-content/uploads/2021/08/CAM-Long_Training-Manual.pdf. Accessed 7 Nov 2023.

[38] Inouye SK, Kosar CM, Tommet D, Schmitt EM, Puelle MR, Saczynski JS (2014). The CAM-S: development and validation of a new scoring system for delirium severity in 2 cohorts. Ann Intern Med.

[39] Meagher D, Adamis D, Leonard M, Trzepacz P, Grover S, Jabbar F (2014). Development of an abbreviated version of the delirium motor subtyping scale (DMSS-4). Int Psychogeriatr.

[40] Garcia Nuñez D, Boettger S, Meyer R, Richter A, Schubert M, Meagher D (2019). Validation and psychometric properties of the German version of the Delirium Motor Subtype Scale (DMSS). Assessment.

[41] Lukas A, Niederecker T, Günther I, Mayer B, Nikolaus T (2013). Self- and proxy report for the assessment of pain in patients with and without cognitive impairment. Z Gerontol Geriatr.

[42] Basler HD, Hüger D, Kunz R, Luckmann J, Lukas A, Nikolaus T (2006). Beurteilung von Schmerz bei Demenz (BESD): Untersuchung zur Validität eines Verfahrens zur Beobachtung des Schmerzverhaltens. Schmerz.

[43] Warden V, Hurley AC, Volicer L (2003). Development and Psychometric Evaluation of the Pain Assessment in Advanced Dementia (PAINAD) Scale. J Am Med Dir Assoc.

[44] Zwakhalen SMG, van der Steen JT, Najim MD (2012). Which score most likely represents pain on the observational PAINAD pain scale for patients with dementia?. J Am Med Dir Assoc.

[45] Kupeli N, Vickerstaff V, White N, Lord K, Scott S, Jones L (2018). Psychometric evaluation of the cohen-mansfield agitation inventory in an acute general hospital setting. Int J Geriatr Psychiatry.

[46] Hülser K (2001). Klinische Diagnostik im Rahmen der Qualitätssicherung [diploma thesis].

[47] Nilges P, Essau C. DASS. Depressions-Angst-Stress-Skalen - deutschsprachige Kurzfassung. 2021. https://www.psycharchives.org/en/item/5bcda8ff-b672-43e3-a6e8-6cbc598bf37e. Accessed 7 Nov 2023.

[48] Richards KC, O'Sullivan PS, Phillips RL (2000). Measurement of sleep in critically ill patients. J Nurs Meas.

[49] Krotsetis S, Richards KC, Behncke A, Köpke S (2017). The reliability of the German version of the Richards Campbell sleep questionnaire. Nurs Crit Care.

[50] epa-CC GmbH. epa-AC. https://www.epa-cc.de/impressum/ (2021). Accessed 07 Nov 2023.

[51] Dichter MN, Roos M, Trigg R, Laporte Uribe F, Dreyer J, Halek M. Bath. Erhebungsinstrument der Subjektiven Lebensqualität bei Demenz (BASQID). Benutzerhandbuch für die deutschsprachige Version 1.0: Anleitung für Nutzerinnen und Nutzer sowie Eigenschaften des Instruments. 2020. https://www.dzne.de/fileadmin/Dateien/editors/images/Standorte/Witten/Projekte/QoL-Dem/BASQID_Benutzerhandbuch_German_final.pdf. Accessed 7 Nov 2023.

[52] Trigg R, Skevington SM, Jones RW (2007). How can we best assess the quality of life of people with dementia? The Bath Assessment of Subjective Quality of Life in Dementia (BASQID). Gerontologist.

[53] Dichter MN, Ettema TP, Schwab CGG, Meyer G, Bartholomeyczik S, Halek M. Benutzerhandbuch für die deutschsprachige QUALIDEM Version 2.0. 2016. https://www.dzne.de/forschung/studien/projekte-der-versorgungsforschung/qol-dem/. Accessed 7 Nov 2023.

[54] Ettema TP, Dröes RM, de Lange J, Mellenbergh GJ, Ribbe MW (2007). QUALIDEM: development and evaluation of a dementia specific quality of life instrument. Scalability, reliability and internal structure. Int J Geriatr Psychiatry..

[55] Suhonen R, Leino-Kilpi H, Välimäki M (2005). Development and psychometric properties of the Individualized care scale. J Eval Clin Pract.

[56] Köberich S, Suhonen R, Feuchtinger J, Farin E (2015). The German version of the individualized care scale - assessing validity and reliability. Patient Prefer Adher.

[57] Nasreddine ZS, Phillips NA, Bédirian V, Charbonneau S, Whitehead V, Collin I (2005). The Montreal Cognitive Assessment, MoCA: a brief screening tool for mild cognitive impairment. J Am Geriatr Soc.

[58] Charlson ME, Pompei P, Ales KL, MacKenzie CR (1987). A new method of classifying prognostic comorbidity in longitudinal studies: development and validation. J Chronic Dis.

[59] Sundararajan V, Henderson T, Perry C, Muggivan A, Quan H, Ghali WA (2004). New ICD-10 version of the Charlson comorbidity index predicted in-hospital mortality. J Clin Epidemiol.

[60] Schmidt SG, Dichter MN, Palm R, Hasselhorn HM (2012). Distress experienced by nurses in response to the challenging behaviour of residents - evidence from German nursing homes. J Clin Nurs.

[61] Slater P, McCance T, McCormack B (2017). The development and testing of the Person-centred Practice Inventory - Staff (PCPI-S). Int J Qual Health Care.

[62] von Dach C, Schlup N, Gschwenter S, McCormack B (2023). German translation, cultural adaptation and validation of the Person-Centred Practice Inventory—Staff (PCPI-S). BMC Health Serv Res.

[63] Möllers T, Perna L, Ihle P, Schubert I, Bauer J, Brenner H (2019). Factors associated with length of stay in hospital patients with and without dementia. J Alzheimers Dis.

[64] Ukoumunne OC, Gulliford MC, Chinn S, Sterne JA, Burney PG (1999). Methods for evaluating area-wide and organisation-based interventions in health and health care: a systematic review. Health Technol Assess.

[65] StataCorp (2019). Stata Statistical Software. Version 16.

[66] Kuss O, Blettner M, Börgermann J (2016). Propensity score: an alternative method of analyzing treatment effects. Dtsch Arztebl Int.

[67] Brookhart MA, Schneeweiss S, Rothman KJ, Glynn RJ, Avorn J, Stürmer T (2006). Variable selection for propensity score models. Am J Epidemiol.

[68] Edmonds WA, Kennedy TD (2017). An applied guide to research designs: quantitative, qualitative, and mixed methods.

[69] Moore GF, Audrey S, Barker M, Bond L, Bonell C, Hardeman W (2015). Process evaluation of complex interventions: medical research council guidance. BMJ.

[70] Grant A, Treweek S, Dreischulte T, Foy R, Guthrie B (2013). Process evaluations for cluster-randomised trials of complex interventions: a proposed framework for design and reporting. Trials.

[71] Ritchie J, Spencer L, Bryman A, Burgess R (1994). Qualitative Data Analysis for Applied Policy Research. Analyzing Qualitative Data.

[72] Mayring P, Fenzl T, Baur N, Blasius J (2019). Qualitative Inhaltsanalyse. Handbuch Methoden der empirischen Sozialforschung.

[73] VERBI Software (2021). MAXQDA. Version 2022.

[74] IBM Corp (2020). IBM SPSS Statistics for windows. Version 27.0.

[75] Mauskopf JA, Paul JE, Grant DM, Stergachis A (1998). The role of cost-consequence analysis in healthcare decision-making. Pharmacoeconomics.

[76] Poitras ME, Maltais ME, Bestard-Denommé L, Stewart M, Fortin M (2018). What are the effective elements in patient-centered and multimorbidity care? A scoping review. BMC Health Serv Res.

[77] McKeown J, Clarke A, Ingleton C, Repper J (2010). Actively involving people with dementia in qualitative research. J Clin Nurs.

[78] Dewing J (2007). Participatory research: a method for process consent with persons who have dementia. Dementia.

