# Supplementary material for: Expanded nursing roles to promote person-centred care for people with cognitive impairment in acute care (ENROLE-acute): study protocol for a controlled clinical trial, process and economic evaluation
Source: BMC Geriatr. 2023 Dec 14;23:858. doi: 10.1186/s12877-023-04560-3 (PMC10722805; doi:10.1186/s12877-023-04560-3)
Supplement: Supplementary file 2 — Additional file 2. Eligibility criteria. [file 12877_2023_4560_MOESM2_ESM.docx]

| **Participants** | **Eligibility criteria** |
| --- | --- |
| People with cognitive impairment | People with cognitive impairment will be included, if all of the following conditions apply:   - Age ≥ 65 years - Unclear duration of hospital stay or planned hospital stay of ≥ 48 hours - Sufficient knowledge of German language to participate - Ability to consent to study participation or existence of a legal representative - Present cognitive impairment or risk for cognitive impairment, indicated by one of the following criteria:   1. Diagnosis of cognitive impairment      - Diagnosis of delirium: F05.*      - Diagnosis of cognitive impairment / dementia: F00.*, F01.*, F02.3, F02.8, F03, U51.*   2. Need for clarification of neurocognitive disorder according to outcome-oriented nursing assessment AcuteCare (epa-AC ©^1^) [1], indicated by confirming at least two of the following six items:      - Vigilance: soporous or coma      - Orientation: to two qualities or less      - Processing information: low ability like processing simple information with increased expenditure of time and support (e.g. repetitions) or no ability      - Everyday competence: low or no ability to act and react to the demands of everyday life on a cognitive level      - Attention: temporarily or continuously impaired      - Drugs that increase the risk of falls/delirium: e.g. antidepressants, benzodiazepine or diuretics   3. High risk for delirium [2] defined as a sum ≥ 3 points:      - Age ≥ 75 years = 2 points      - Hearing impairment = 2 points      - Psychotropic drugs (at least one chronically prescribed drug) = 1 point      - Surgery during hospital stay = 1 point   People with cognitive impairment will be excluded, if one of the following conditions applies:   - Cognitive impairment due to   - Delirium tremens (F10.4)   - Delirium because of other substance withdrawal   - Psychiatric diagnosis (F10.-*, F20.-*, F04, F06.-*, F07.-*, F09, oder F25.-*)   - Traumatic brain injuries (S06.*)   - Dementia associated with diseases classified elsewhere (F02.0, F02.1, F02.2, F02.4) - Patients in the terminal phase (end of life) |
| Relatives | Relatives are persons who, according to the patient, have a close social relationship with him/her. Relatives will be included if they have visited the person with cognitive impairment at least once during the hospital stay on the project ward. |
| Expanded Practice Nurses | Expanded practice nurses will be included in the project, if all of the following conditions apply:   - Qualified at level 6 of European Qualifications Framework, indicated by:   - academic degree in nursing (at least Bachelor’s degree) or   - three years vocational training and two years of further training according to recommendations of German Hospital Association or   - three years vocational training and expanded competencies according to ward manager through predefined criteria [3] - Minimum employment of 75 % - Willingness to participate in the training and the internship - Good English language skills - Good communication and interaction skills - Enthusiasm for and experience with person-centred care for people with cognitive impairment in the hospital setting |
| Wards | Wards will be included, if all of the following conditions apply:   - Prevalence of at least 30 people with cognitive impairment per month according to data   from hospital controlling   - Peripheral wards   Wards will be excluded, if one of the following conditions apply:   - Intensive care units - Intermediate care units - psychiatric, palliative and paediatric wards |
| Interprofessional team | Members of the interprofessional team will be included, if all of the following conditions apply:   - Working on the project ward during the time of data collection - Working on the project ward as registered nurse (European Qualifications Framework level 4), nurse assistant or physician   Members of the interprofessional team will be excluded, if one of the following conditions applies:   - Working on the project ward as therapist, service or administrative staff |
| Ward managers | Ward managers will be included if all of the following conditions apply:   - Working on the project ward during time of data collection |
| Nursing management | Members of nursing management are nursing managers at level of the hospital responsible for the participating wards. |
| Nursing development unit | Members of the nursing development unit are nursing scientists responsible for nursing development at the hospital. |

^1^ With outcome-oriented nursing assessment AcuteCare (epa-AC ©) the patient's abilities and impairments are assessed daily by nurses using the four-level epa point system and recorded in electronic form.

1. epa-CC GmbH. epa-AC. https://www.epa-cc.de/impressum/ (2021). Accessed 07 Nov 2023.
2. Zucchelli A, Apuzzo R, Paolillo C, Prestipino V, De Bianchi S, Romanelli G, et al. Development and validation of a delirium risk assessment tool in older patients admitted to the Emergency Department Observation Unit. Aging Clin Exp Res. 2021;33(10):2753-8.
3. Müskens W, Lübben S. Generische Deskriptoren für informelles Lernen. Entwicklung eines Instruments zur Niveaubewertung bei der Anrechnung von Kompetenzen auf Hochschulstudiengänge. 2018. https://uol.de/fileadmin/user_upload/anrechnungsprojekte/Download/Artikel_Generische_Deskriptoren_GDIL.pdf. Accessed 7 Nov 2023.
